# Factors associated with sexually transmitted infections among reproductive age women in Ivory Coast: evidenced by 2021 Ivory Coast Demographic and Health Survey

**DOI:** 10.3389/fgwh.2025.1490762

**Published:** 2025-05-02

**Authors:** Gosa Mankelkl, Beletu Kinfe

**Affiliations:** ^1^ Department of Biomedical Sciences, College of Medicine and Health Science, Wollo University, Dessie, Ethiopia; ^2^ Department of Occupational Health and Safety, College of Medicine and Health Science, Wollo University, Dessie, Ethiopia

**Keywords:** prevalence, sexually transmitted infections, Ivory Coast, reproductive, women

## Abstract

**Background:**

Globally, sexually transmitted infections (STIs) continue to be a major public health problem. STIs are a major cause of morbidity and mortality in many developing countries due to their effects on reproductive and child health and their role in spreading HIV infection. This study ' to determine the factors associated with STIs among women in Ivory Coast using data from the 2021 Ivory Coast Demographic and Health Survey (DHS).

**Methods:**

A total of 14,877 women from the 2021 Ivory Coast Demographic and Health Survey participated in this study. The Ivory Coast DHS employed a community-based cross-sectional study design for data collection. STATA version 14 was used for data extraction, recoding, descriptive analysis, and analytical analysis. Bivariable analysis was performed to identify factors for multivariable analysis. In the multivariable analysis, factors with a significance level of *P* < 0.05 were considered significant predictors of STIs among reproductive-age women. Finally, frequency, percentage, and odds ratios with a 95% confidence interval were reported.

**Result:**

This study includes a total weighted sample of 14,877 women from the 2021 Ivory Coast Demographic and Health Survey. The prevalence of STIs among reproductive-age women in the last 12 months was 6.82%, with a 95% CI (6.42, 7.23). The results of the multivariate analysis showed that among women, STIs were statistically and significantly associated with age range of 20–24 years [adjusted odds ratio (AOR): 1.558, 95% CI: (1.108, 2.359); *P* = 0.011], 25–29 years [AOR: 1.523, 95% CI: (1.089, 2.129); *P* = 0.014], and 30–34 years [AOR: 1.655, 95% CI: (1.191, 2.300); *P* = 0.003]; living in Denguele [AOR:2.138, 95% CI: (1.328, 3.439); *P* = 0.002], Montagnes [AOR: 2.930, 95% CI: (1.909, 4.497); *P* = 0.0001], and Zanzan [AOR: 2.330, 95% CI: (1.476, 3.679); *P* = 0.0001]; being married [AOR: 0.705, 95% CI: (0.520, 0.975); *P* = 0.034]; being Muslim [AOR: 0.785, 95% CI: (0.621, 0.993); *P* = 0.011]; listening to radio at least once a week [AOR: 1.524, 95% CI: (1.241, 1.871); *P* = 0.0001]; watching television less than once a week [AOR: 1.649, 95% CI: (1.156, 2.352); *P* = 0.006]; using the internet almost every day [AOR: 1.359, 95% CI: (1.081, 1.708); *P* = 0.008]; having a history of a terminated pregnancy [AOR: 1.170, 95% CI: (1.017, 1.376); *P* = 0.043]; using modern contraceptives [AOR: 1.213, 95% CI: (1.032,1.427); *P* = 0.0001]; and being tested for HIV [AOR: 1.342, 95% CI: (1.149, 1.569); *P* = 0.0001].

**Conclusion and recommendations:**

This study found that nearly seven out of a hundred reproductive-age women in Ivory Coast had sexually transmitted infections, influenced by factors such as age group, region, religion, marital status, media exposure (reading magazines, watching television, and using the internet), history of a terminated pregnancy, and contraceptive utilization. Therefore, healthcare providers and policymakers should focus on these specific predictors to reduce STIs among reproductive-age women.

## Introduction

The term “sexually transmitted infections” (STIs) refers to a pathogen that causes infection through sexual contact (1). Naturally, STIs affect individuals within partnerships and larger sexual networks, and in turn, the general population (2, 3). STIs are a global public health issue, especially in developing countries (4), contributing significantly to morbidity (the rate of disease) and mortality (the rate of death) in the population (5). The World Health Organization (WHO) reported 374 million new cases of curable STIs annually in 2021 (6), with sub-Saharan Africa accounting for 40% of the global burden (7). Currently, over a million STIs are acquired daily, with approximately half a billion new cases of STIs reported annually worldwide (8, 9). In Côte d’Ivoire, epidemiological studies have revealed that over 10% of women have STIs (10). In addition, a cross-sectional study of women in Côte d’Ivoire found that 5.5% had chlamydial infections and 3.7% had gonococcal infections (11).

The greater impact of STIs on women compared with men is partly due to the female anatomy. The female urogenital system is more exposed and vulnerable to STIs compared with the male urogenital anatomy, particularly because the vaginal mucosa is thin, delicate, and easily penetrated by infectious agents (12). The cervix located at the distal end of the vagina connects to the upper genital tract, including the uterus, endometrium, fallopian tubes, and ovaries. STIs can cause a variety of symptoms and complications across different parts of the female reproductive tract, including genital ulcer disease, vaginitis, pelvic inflammatory disease, infertility, cervical cancer, and pregnancy complications (13), and drug resistance is a major threat to efforts aimed at reducing the burden of STIs worldwide (14).

Several studies demonstrated that STIs among women are associated with sociodemographic, socioeconomic, and geographic factors such as age groups, marital status, sex of household head, place of residence, education status, religion, and media exposure (15, 16).

To improve the quality of life and accomplish the goal of eradicating new HIV infections, the Ivory Coast government has endorsed a number of international commitments and strategies (17–20). However, while HIV prevention and treatment have received more public health attention in recent years than STIs, other STIs have received less attention. Although several studies have been conducted in the Ivory Coast to address this issue in different settings (19, 21–25), there is no updated and reliable national-level evidence on the factors associated with STIs among reproductive-age women. Furthermore, most of these studies lack representativeness, as they were conducted in specific regions rather than at the national level. Therefore, this study aimed to identify the factors associated with STIs among women in Ivory Coast at the national level using the Ivory Coast Demographic and Health Survey (DHS). Furthermore, policymakers and other stakeholders may prefer national-level findings, which serve as a foundation for designing and implementing appropriate intervention programs aimed at reducing the rate of STIs among women. In addition, this study also serves as a valuable reference for future research in this field.

## Methods and materials

### Study setting and period

Ivory Coast is a country in West Africa, located between 4°30′ and 10°30′ north latitude. It extends over an area of 322,462 km^2^ and is bordered to the north by Mali and Burkina Faso, to the west by Liberia and Guinea, to the east by Ghana, and to the south by the Atlantic Ocean. According to the 2021 population census, the country had 29,389,150 inhabitants, with 45% aged <18 years old. Women aged 15–49 represented 24% of the total population. The National Institute of Statistics (INS) carried out the 2021 Ivory Coast Demographic and Health Survey from 8 September to 30 December 2021, with technical assistance from the International Classification of Functioning, Disability, and Health (ICF) and specialized departments of the Ministry of Health, Public Hygiene, and Universal Health Coverage (26, 27).

### Data source/data extraction

The data used in this analysis were obtained from the 2021 Ivory Coast Demographic and Health Survey, accessible through the DHS portal at https://dhsprogram.com/data/dataset_admin/index.cfm. Permission was obtained through an online request by explaining the purpose of the study (26, 27).

### Study design

This study employed a community-based cross-sectional design, as the DHS used this design to collect data.

### Sampling procedure

**Figure 1:**
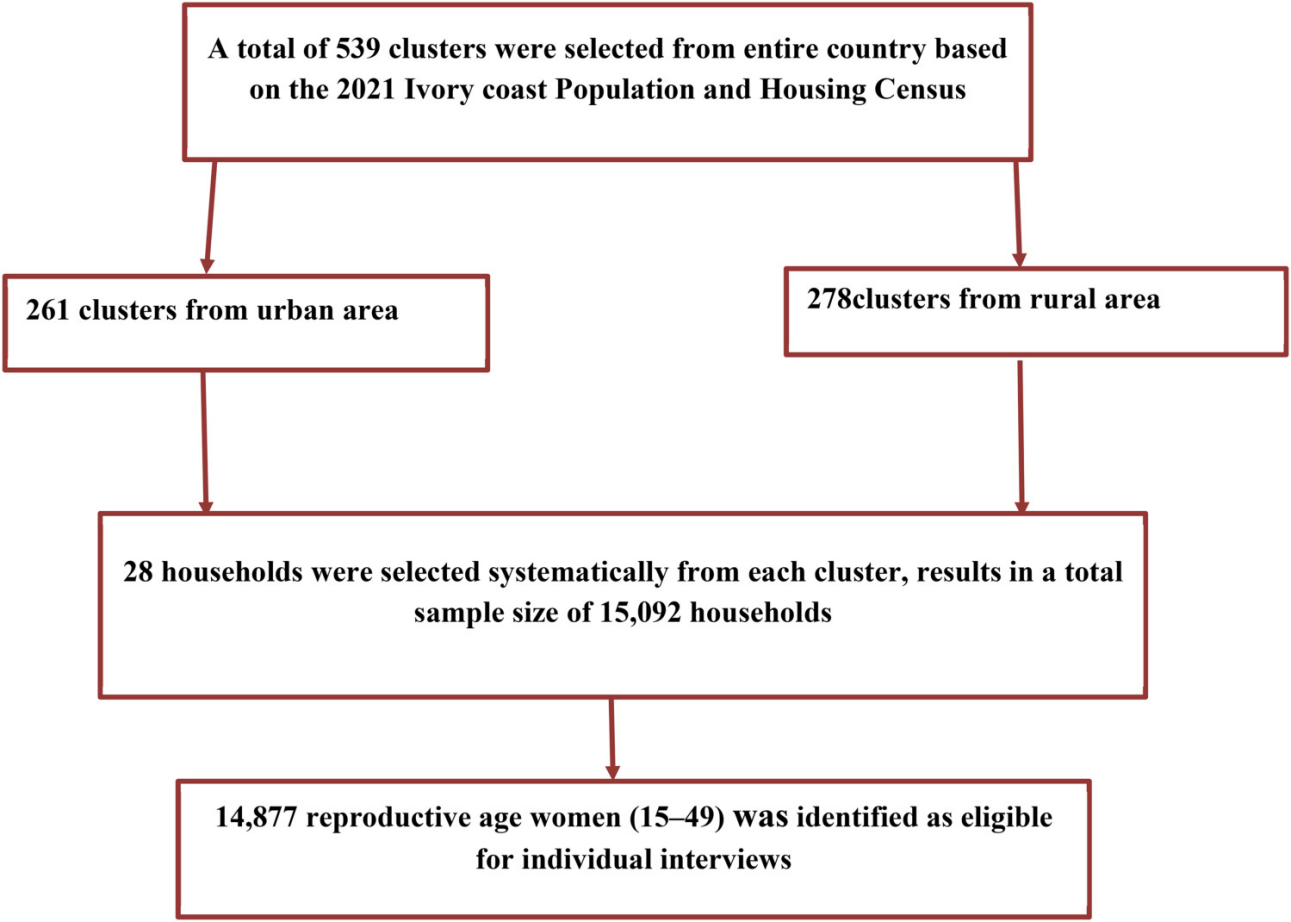
The sampling process of reproductive-age women (27).

To ensure representativeness, the sampling process for the 2021 Ivory Coast Demographic and Health Survey was created, taking into account the country's 14 administrative districts as well as both urban and rural residential areas. The selection procedure for drawing the sample had two stages. At the first level, 539 clusters were selected for investigation, 261 of which were found in urban areas and 278 in rural areas. The National Institute of Statistics conducted a census mapping in 2019 to create a list of clusters, which served as the basis for systematically selecting clusters with a probability proportional to household size. This process was carried out prior to the 2021 Population and Housing Census (RGPH) (26, 27). At the second level, a sample of 15,092 households was selected at a rate of 28 households per cluster, 7,308 of which were located in urban areas and 7,784 in rural areas (Figure 1).

### Study population

All women aged 15–49 who were either long-term residents of the selected households or guests who had spent the night before the survey were eligible to be interviewed. Data were collected from 14,877 reproductive-age women through face-to-face interviews using a standardized women's questionnaire. In this study, STIs among reproductive-age women were the outcome variable (27).

### Study variables

The **outcome variable** for this study was STIs among women. This variable has a binary result (yes or no). Women were asked whether they had a disease they had acquired a disease through sexual contact in the past 12 months. If they had not acquired STIs in the last 12 months, responses were labeled as “no” and coded as “0”; otherwise, responses were labeled as “yes” and coded as “1”.

The independent variables included age group, place of residence, region, religion, educational status, marital status, sex of the household head, wealth index, media exposure (reading magazines, watching television, and using the internet), history of a terminated pregnancy, had health insurance, contraceptive utilization, being tested for HIV, currently working, number of unions, community media exposure, and community wealth quantile (Table 1).

**Table 1:** The description of the independent variables.

Lists of variables	Definitions/categories
Age group (age of mother in years)	The age of the mother was coded as 15–19, 20–24, 25–29, 30–34, 35–39, 40–44, and 45–49 in the IR file of the Ivory Coast Demographic and Health Survey
Place of residence	Places of residence in the 2021 Ivory Coast Demographic and Health Survey were grouped as urban and rural
Region	Region in the 2021 Ivory Coast Demographic and Health Survey were categorized into 14 administrative regions. We used it as it is in our analysis
Religion	Religion was coded as Muslim, Catholic, Methodist, Evangelical, other Christian religion, Animist, other, and no religion in the Ivory Coast Demographic and Health Survey. Depending on the number of participants, we were regrouped into Muslim, Catholic, Evangelical, and others (Methodist, other Christian religion, Animist, other, and no religion)
Educational status	The level of education in the 202I Ivory Coast Demographic and Health Survey was categorized into four groups such as attending no education, primary education, secondary education, and higher education
Sex of the household head	The sex of household heads was categorized into male-headed household and female-headed household heads
Wealth index	The combined wealth index in the 202I Ivory Coast Demographic and Health Survey was categorized into poorest, poorer, middle, richer, and richest. So, we used it in our analysis as it is.
Frequency of reading magazines, listening to the radio, watching television, and reading using the internet	The frequency of reading magazines; the frequency of listening to the radio, the frequency of watching television, and the frequency of using the internet were categorized into four groups such as not at all, less than once a week, at least once a week, and almost every day respectively in the 2021 Ivory Coast Demographic and Health Survey
History of a terminated pregnancy	Reproductive-age women who had no history of a terminated pregnancy were coded as “no,” and reproductive-age women who had a history of a terminated pregnancy were coded as “yes” in the IR file of the 202I Ivory Coast Demographic and Health Survey
Contraceptive utilization	In the IR file of the 202I Ivory Coast Demographic and Health Survey, it is coded as no method, folkloric method, traditional method, and modern method. We recategorized it into no method, modern method, and other methods (folkloric method, traditional method) due to the fact that few participants used folkloric and traditional methods
Age at first sex	In the IR file of the 202I Ivory Coast Demographic and Health Survey, age at first sex was represented by a numerical value. Then, we recategorized it into three groups such as had no sex; sex at the age of 18 years and below; and sex above 18 years old.
Had ever heard about STIs; being tested for HIV; had health insurance; and currently working	In the 202I Ivory Coast Demographic and Health Survey categorized the variables such as had ever heard about STIs; being tested for HIV; had health insurance; and currently working were the dichotomous variables categorized into no and yes.
Community media exposure or media exposure (radio, TV, internet)	The frequency of listening to radio, the frequency of watching television, and the frequency of using the internet were coded as not at all, less than once a week, and at least once a week, respectively. We recategorized into “no” and “yes.” Then, participants who had no exposure to media at all (reading magazines, watching television, and using the internet) were coded “no,” and participants who had media exposure (reading magazines, watching television, and using the internet) less than once a week, at least once a week, and almost every day were coded “yes.” Women who had at least one or more media exposure from the three (reading magazines, listening to the radio, watching television) labeled as “yes,” and women who had not at least one media exposure from the three (reading magazines, listing to the radio, watching television) was coded as “no.”
Community wealth quantile	The wealth index combined and wealth index for urban/rural have five categories, i.e., poorest, poorer, middle, richer, and richest. We recategorized it into three subgroups, i.e., “poorest and poorer” grouped as poor; middle grouped as it is; and “richer and richest” grouped as rich. Subsequently, we merged the wealth index combined and wealth index for urban/rural; if participants were poor in both the wealth index combined and wealth index for urban/rural, they were considered as poor; if participants were middle in one of the two groups, they were considered to belong in the middle wealth quantile; and if participants were rich at least in one of the two the groups (wealth index combined and wealth index for urban/rural), they were considered as rich.

### Data management and analysis

Data extraction, recoding, descriptive analysis, and analytical analysis were all performed using STATA version 14. We carried out weighting by dividing the total number of women in the entire country by the appropriate survey sampling proportion and the standard weights of the women. Bivariate analysis was first employed to identify the potential factors associated with STIs among reproductive-age women. This initial analysis assessed the relationship between each independent variable and the outcome variable (STIs), allowing for the identification of factors that showed significant associations with STIs. Variables that demonstrated a significant relationship with the outcome in the bivariate analysis, indicated by *P* < 0.05, were included in the multivariate analysis. The multivariate analysis was then conducted to examine the independent effect of each selected variable while controlling for potential confounding factors. Only those variables with *P* < 0.05 in the multivariate analysis were considered significant predictors of STIs. Finally, the percentage and odd ratios were provided, together with their 95% confidence intervals.

### Ethical consideration

This study used secondary data from the Demographic and Health Survey (DHS), which did not require ethical review or participant consent. The data ensured confidentiality, adhering to DHS privacy protocols. The integrity of the data was preserved, as it was originally collected following ethical guidelines. Data use complied with DHS guidelines and respected the informed consent provided by participants. No new data were collected, and there was no direct participant interaction. The findings were presented responsibly to minimize misinterpretation.

## Results

### Sociodemographic characteristics of the participants

A total of 14,877 women participated in this investigation. Approximately 3,089 participants (20.76%) were in the 15–19 age range; 7,457 (50.12%) were rural residents; 1,416 (9.52%) were from the Abidjan region; 7,994 (53.73%) were non-educated; 6,923 (46.53%) were Muslims; and 5,218 (35.07%) were married. Households headed by men accounted for 11,555 (77.67%); 3,439 (23.12%) were in the middle wealth quantile; 13,449 (90.40%) did not read at all; 9,646 (64.84%) did not listen to the radio. Television was watched at least once a week by 7,147 (48.04%), and 11,356 (76.33%) did not use the internet at all. In terms of reproductive health, 2,942 (19.78%) had a history of a terminated pregnancy; 2,652 (17.83%) utilized modern contraceptives; and 11,637 (77.22%) had their first sex at the age of 18 and below. Health insurance coverage was reported by 14,061 (94.52%); 8,381 (56.34%) were never tested for HIV; 13,185 (88.63%) had ever heard of STIs; 8,442 (56.75%) were currently working; 9,313 (62.60%) had single union; 10,302 (69.25%) had community media exposure; and 6,179 (41.53%) belonged to the rich wealth quantile (Table 2).

**Table 2:** Sociodemographic characteristics of women in Ivory Coast in 2021.

Characteristics (*n* = 14,877)	Categories	Had any STI		Frequency	Percentage
		No	Yes		
Age groups	15–19	2,966	123	3,089	20.76
	20–24	2,389	223	2,612	17.56
	25–29	2,127	194	2,321	15.60
	30–34	2,080	200	2,280	15.33
	35–39	1,872	130	2,002	13.46
	40–44	1,452	89	1,541	10.36
	45–49	977	55	1,032	6.94
Place of residence	Urban	6,839	581	7,420	49.88
	Rural	7,024	433	7,457	50.12
Region	Abidjan	1,314	102	1,416	9.52
	Yamoussoukro	803	64	867	5.83
	Bas-Sassandra	1,130	47	1,177	7.91
	Comoe	770	78	848	5.70
	Denguele	1,004	91	1,095	**7** **.** **36**
	Goh-Djiboua	928	54	982	6.60
	Lacs	907	51	958	6.44
	Lagunes	951	74	1,025	6.89
	Montagnes	1,023	161	1,184	7.96
	Sassandra Marahoue	1,300	40	1,340	9.01
	Savanes	1,026	50	1,076	7.23
	Vallee du Bandama	947	55	1,002	6.74
	Woroba	998	39	1,037	6.97
	Zanzan	762	108	870	5.85
Educational status	No education	7,542	452	7,994	53.73
	Primary	2,518	223	2,741	18.42
	Secondary	3,308	282	3,590	24.13
	Higher	495	57	552	3.71
Religion	Muslim	6,534	389	6,923	46.53
	Catholic	2,218	200	2,418	16.25
	Evangelical	3,073	269	3,342	22.46
	Other	2,038	156	2,194	14.75
Marital status	Never in union	4,211	289	4,500	30.25
	Married	4,891	327	5,218	35.07
	Living with partner	4,076	330	4,406	29.62
	Other	685	68	753	5.06
Sex of the household head	Male	10,811	744	11,555	77.67
	Female	3,052	270	3,322	22.33
Wealth index	Poorest	2,987	148	3,135	21.07
	Poorer	2,950	192	3,142	21.12
	Middle	3,195	244	3,439	23.12
	Richer	2,527	221	2,748	18.47
	Richest	2,204	209	2,413	16.22
Frequency of reading newspapers	Not at all	12,569	880	13,449	90.40
	Less than once a week	26	78	904	6.08
	At least once a week	468	56	524	3.52
Frequency of listening to the radio	Not at all	9,131	515	9,646	64.84
	Less than once a week	2,686	272	2,958	19.88
	At least once a week	2,046	227	2,273	15.28
Frequency of watching television	Not at all	5,189	241	5,430	36.50
	Less than once a week	2,092	208	2,300	15.46
	At least once a week	6,582	565	7,147	48.04
Frequency of using the Internet	Not at all	10,710	646	11,356	76.33
	Less than once a week	366	36	402	2.70
	At least once a week	1,071	130	1,201	8.07
	Almost every day	1,716	202	1,918	12.89
History of a terminated pregnancy	No	11,191	744	11,935	80.22
	Yes	2,672	270	2,942	19.78
Contraceptive utilization	No method	11,072	713	11,785	79.22
	Modern method	2,380	272	2,652	17.83
	other method	411	29	440	2.96
Age at first sex	Not had sex	1,663	0	1,663	11.18
	At the age of 18 years and below	10,751	886	11,637	78.22
	Above 18 years old	1,449	128	1,577	10.60
Had ever heard about STIs	No	1,692	0	1,692	11.37
	Yes	12,171	1,014	13,185	88.63%
Being tested for HIV	No	7,979	402	8,381	56.34
	Yes	5,884	612	6,496	43.66
Had health insurance	No	13,119	942	14,061	94.52
	Yes	744	72	816	5.48
Currently working	No	6,029	406	6,435	43.25
	Yes	7,834	608	8,442	56.75
Number of unions	Never	4,211	289	4,500	30.25
	Once	8,671	642	9,313	62.60
	More than once	981	83	1,064	7.15
Community media exposure	No	4,384	191	4,575	30.75
	Yes	9,479	823	10,302	69.25
Community wealth quantile	Poor	3,685	202	3,887	26.13
	Middle	4,488	323	4,811	32.34
	Rich	5,690	489	6,179	41.53

### Prevalence of sexually transmitted infections

The prevalence of STIs among women in the 12 months preceding the survey was 6.82%, with a 95% CI (6.42–7.23). The highest prevalence of STIs was observed in the Montagnes region (161 cases, 15.88%), followed by Zanzan (108 cases, 10.65%) and Abidjan (102 cases, 10.06%). In contrast, the lowest prevalence was recorded in Bas-Sassandra (47 cases, 4.64%), Sassandra-Marahoué (40 cases, 3.94%), and Woroba (39 cases, 3.85%) (Table 3).

**Table 3:** Prevalence of sexually transmitted infections among reproductive-age women in Ivory Coast in 2021.

List of variables (*n* = 14,877)	Categories	Had any STIs	Frequency (%)
Region	Abidjan	No	1,314 (9.48)
		Yes	102 (10.06)
	Yamoussoukro	No	803 (5.79)
		Yes	64 (6.31)
	Bas-Sassandra	No	1,130 (8.15)
		Yes	47 (4.64)
	Comoe	No	770 (5.55)
		Yes	78 (7.69)
	Denguele	No	1,004 (7.24)
		Yes	91 (8.97)
	Goh-Djiboua	No	928 (6.69)
		Yes	54 (5.33)
	Lacs	No	907 (6.54)
		Yes	51 (5.03)
	Lagunes	No	951 (6.86)
		Yes	74 (7.30)
	Montagnes	No	1,023 (7.38)
		Yes	161 (15.88)
	Sassandra Marahoue	No	1,300 (9.38)
		Yes	40 (3.94)
	Savanes	No	1,026 (7.40)
		Yes	50 (4.93)
	Vallee du Bandama	No	947 (6.83)
		Yes	55 (5.42)
	Woroba	No	998 (7.20)
		Yes	39 (3.85)
	Zanzan	No	762 (5.50)
		Yes	108 (10.65)
Ivory Coast	Total	No	13,863 (93.18), CI (92.76 −93.57)
		Yes	1,014 (6.82), CI (6.42–7.23)

### Bivariable analysis on factors analysis associated with sexually transmitted infections

The results of the bivariable analysis showed that STIs among women were statistically and significantly associated with the following factors: age range of 20–24 years [crude odds ratio (COR): 1.770, 95% CI: (1.254, 2.359); *P* = 0.001], 25–29 years [COR: 1.734, 95% CI: (1.258, 2.391); *P* = 0.001], and 30–34 years [COR: 1.841, 95% CI: (1.337, 2.534); *P* = 0.0001]; living in Montagnes [COR: 2.027, 95% CI: (1.561, 2.632); *P* = 0.0001]; living in an urban area [COR: 1.378, 95% CI: (1.211, 1.567); *P* = 0.0001]; having no education [COR: 0.538, 95% CI: (0.391, 0.739); *P* = 0.0001]; religion [COR: 0.538, 95% CI: (0.391, 0.739); *P* = 0.0001]; being married [COR: 0.673, 95% CI: (0.512, 0.885); *P* = 0.005]; being the head of a female-headed household [COR: 1.285, 95% CI: (1.112, 1.485); *P* = 0.001]; being in the richest wealth category [COR: 1.913, 95% CI: (1.539, 2.379); *P* = 0.0001]; having a history of a terminated pregnancy [COR: 1.521, 95% CI: (1.303, 1.774); *P* = 0.0001]; having health insurance [COR: 1.347, 95% CI: (1.048, 1.731); *P* = 0.0001]; being tested for HIV [COR: 2.064, 95% CI: (1.812, 2.351); *P* = 0.0001]; being currently employed [COR: 1.152, 95% CI: (1.011, 1.312); *P* = 0.032]; having community media exposure [COR: 1.992, 95% CI: (1.695, 2.341); *P* = 0.0001]; and being wealthy [COR: 1.567, 95% CI: (1.323, 1.856); *P* = 0.0001]. The above-stated variables were considered for multivariable analysis (Table 4).

**Table 4:** Bivariable analysis on factors associated with STIs among women in Ivory Coast in 2021.

Characteristics (*n* = 14,877)	Categories	Had any STI		COR with 95% CI; *P*-value
		No	Yes	
Age groups	15–19	2,966	123	0.745 (0.531, 1.043); 0.087
	20–24	2,389	223	1.720 (1.254, 2.359); 0.001
	25–29	2,127	194	1.734 (1.258, 2.391); 0.001
	30–34	2,080	200	1.841 (1.337, 2.534); 0.0001
	35–39	1,872	130	1.338 (0.956, 1.874); 0.089
	40–44	1,452	89	1.174 (0.821, 1.680); 0.377
	45–49	977	55	1
Place of residence	Urban	6,839	581	1.378 (1.211, 1.567); 0.0001
	Rural	7,024	433	1
Region	Abidjan	1,314	102	1
	Yamoussoukro	803	64	1.026 (0.742, 1.420); 0.873
	Bas-Sassandra	1,130	47	0.535 (0. 375, 0.763); 0.001
	Comoe	770	78	1.304 (0.959, 1.775); 0.090
	Denguele	1,004	91	1.167 (0.869, 1.567); 0.302
	Goh-Djiboua	928	54	0.749 (0.533, 1.053); 0.097
	Lacs	907	51	0.724 (0.512, 1.024); 0.068
	Lagunes	951	74	1.002 (0.734, 1.367); 0.988
	Montagnes	1,023	161	2.027 (1.561, 2.632); 0.0001
	Sassandra Marahoue	1,300	40	0.396 (0.272, 0.575); 0.0001
	Savanes	1,026	50	0.627 (0.443, 0.889); 0.009
	Vallee du Bandama	947	55	0.748 (0.533, 1.049); 0.093
	Woroba	998	39	0.503 (0.344, 0.734); 0.0001
	Zanzan	762	108	1.825 (1.373, 1.567); 0.0001
Educational status	No education	7,542	452	0.538 (0.391, 0.739); 0.0001
	Primary	2,518	223	0.787 (0.566, 1.094); 0.154
	Secondary	3,308	282	0.736 (0.534, 1.013); 0.060
	Higher	495	57	1
Religion	Muslim	6,534	389	0.733 (0.586, 0.913); 0.006
	Catholic	2,218	200	1.136 (0.896, 1.439); 0.291
	Evangelical	3,073	269	1.124 (0.901, 1.403); 0.299
	Other	2,038	156	1
Marital status	Never in union	4,211	289	0.691 (0.524, 0.911); 0.009
	Married	4,891	327	0.673 (0.512, 0.885); 0.005
	Living with partner	4,076	330	1.815 (0.620, 1.071); 0.144
	Other	685	68	1
Sex of the household head	Male	10,811	744	1
	Female	3,052	270	1.285 (1.112, 1.485); 0.001
Wealth index	Poorest	2,987	148	1
	Poorer	2,950	192	1.313 (1.053, 1.637); 0.015
	Middle	3,195	244	1.541 (1.249, 1.901); 0.0001
	Richer	2,527	221	1.765 (1.423, 2.188); 0.0001
	Richest	2,204	209	1.913 (1.539, 2.379); 0.0001
Frequency of reading newspapers	Not at all	12,569	880	1
	Less than once a week	26	78	1.348 (1.058, 1.718); 0.015
	At least once a week	468	56	1.709 (1.284, 2.273); 0.0001
Frequency of listening to the radio	Not at all	9,131	515	1
	Less than once a week	2,686	272	1.795 (1.540, 2.092); 0.0001
	At least once a week	2,046	227	1.967 (1.670, 2.316); 0.0001
Frequency of watching television	Not at all	5,189	241	1
	Less than once a week	2,092	208	2.140 (1.766, 2.594); 0.0001
	At least once a week	6,582	565	1.848 (1.582, 2.158); 0.0001
Frequency of using the internet	Not at all	10,710	646	1
	Less than once a week	366	36	1.630 (1.147, 2.317); 0.006
	At least once a week	1,071	130	2.012 (1.649, 2.454); 0.0001
	Almost every day	1,716	202	1.951 (1.653, 2.304); 0.0001
History of a terminated pregnancy	No	11,191	744	1
	Yes	2,672	270	1.521 (1.303, 1.774); 0.0001
Had health insurance	No	13,119	942	1
	Yes	744	72	1.347 (1.048, 1.731); 0.020
Contraceptive utilization	No method	11,072	713	1
	Modern method	2,380	272	1.827 (1.563, 2.136); 0.0001
	other method	411	29	1.095 (0.746, 1.608); 0.641
Being tested for HIV	No	7,979	402	1
	Yes	5,884	612	2.064 (1.812, 2.351); 0.0001
Currently working	No	6,029	406	1
	Yes	7,834	608	1.152 (1.011, 1.312); 0.032
Number of unions	Never	4,211	289	1
	Once	8,671	642	1.078 (0.934, 1.245); 0.300
	More than once	981	83	1.232 (0.956, 1.588); 0.106
Community media exposures	No	4,384	191	1
	Yes	9,479	823	1.992 (1.695, 2.341); 0.0001
Community wealth quantile	Poor	3,685	202	1
	Middle	4,488	323	1.312 (1.095, 1.573); 0.003
	Rich	5,690	489	1.567 (1.323, 1.856); 0.0001

COR, crude odds ratio; CI, confidence interval; others (widowed, divorced, and no longer living together).

### Multivariable analysis on factors associated with sexually transmitted infections

The results of the multivariate analysis showed that among women, STIs were statistically and significantly associated with age group, region, religion, marital status, frequency of reading magazines, frequency of listening to radio, frequency of watching television, frequency of using the internet, history of a terminated pregnancy, contraceptive utilization, and being tested for HIV. The findings of the study demonstrated that the odds of STIs between the age range of 20–24 years [adjusted odds ratio (AOR): 1.558, 95% CI: (1.108, 2.359); *P* = 0.011], 25–29 years [AOR: 1.523, 95% CI: (1.089, 2.129); *P* = 0.014], and 30–34 years [AOR: 1.655, 95% CI: (1.191, 2.300); *P* = 0.003] were more likely compared to women whose ages were between 45 and 49 years old. The odds of STIs among women who were living in Denguele [AOR: 2.138, 95% CI: (1.328, 3.439); *P* = 0.002], Montagnes [AOR: 2.930, 95% CI: (1.909, 4.497); *P* = 0.0001], and Zanzan [AOR: 2.330, 95% CI: (1.476, 3.679); *P* = 0.0001] were more likely relative to women who were living in Abidjan. The odds of STIs among women who were married were 0.712 times less likely [AOR: 0.705, 95% CI: (0.520, 0.975); *P* = 0.034] relative to women who were others (divorced, widowed, and not living together). The odds of STIs among women who were Muslim were 0.785 (0.621, 0.993) times less likely [AOR: 0.785, 95% CI: (0.621, 0.993); *P* = 0.011] relative to other religions.

The odds of STIs among reproductive-age women who listened to radio at least once a week [AOR: 1.524, 95% CI: (1.241, 1.871); *P* = 0.0001]; watching television less than once a week [AOR: 1.649, 95% CI: (1.156, 2.352); *P* = 0.006]; and using the internet almost every day [AOR: 1.359, 95% CI: (1.081, 1.708); *P* = 0.008] were more likely relative to their counterparts. The odds of STIs among women with a history of a terminated pregnancy were 1.170 times more likely [AOR: 1.170, 95% CI: (1.017, 1.376); *P* = 0.043] compared to their counterparts. The odds of STIs among women who use modern contraceptives were 1.213 times more likely [AOR: 1.213, 95% CI: (1.032, 1.427); *P* = 0.0001] compared to women who didn’t use contraceptives. The odds of STIs among women who were tested for HIV were 1.342 times more likely [AOR: 1.342, 95% CI: (1.149, 1.569); *P* = 0.0001] compared to women who were not tested for HIV (Table 5).

**Table 5:** Multivariable analysis on factors associated with STIs among women in Ivory Coast in 2021.

Characteristics (*n* = 14,877)	Categories	Had any STI		AOR with 95% CI; *P*-value
		No	Yes	
Age groups	15–19	2,966	123	1.259 (.854, 1.857); 0.244
	20–24	2,389	223	1.558 (1.108, 2.359); 0.011
	25–29	2,127	194	1.523 (1.089, 2.129); 0.014
	30–34	2,080	200	1.655 (1.191, 2.300); 0.003
	35–39	1,872	130	1.211 (.859, 1.706); 0.273
	40–44	1,452	89	1.148 (0.799, 1.649); 0.453
	45–49	977	55	1
Place of residence	Urban	6,839	581	1.127 (0.865, 1.467); 0.375
	Rural	7,024	433	1
Region	Abidjan	1,314	102	1
	Yamoussoukro	803	64	1.013 (0.632, 1.624); 0.955
	Bas-Sassandra	1,130	47	0.697 (0.427, 1.140); 0.151
	Comoe	770	78	1.563 (0.989, 2.468); 0.055
	Denguele	1,004	91	2.138 (1.328, 3.439); 0.002
	Goh-Djiboua	928	54	0.903 (0.554, 1.471); 0.683
	Lacs	907	51	0.762 (0.463, 1.255); 0.287
	Lagunes	951	74	1.223 (0.773, 1.934); 0.389
	Montagnes	1,023	161	2.930 (1.909, 4.497); 0.0001
	Sassandra Marahoue	1,300	40	0.551 (0.332, 0.914); 0.021
	Savanes	1,026	50	0.986 (0.596, 1.633); 0.959
	Vallee du Bandama	947	55	1.038 (0.642, 1.678); 0.878
	Woroba	998	39	0.891 (0.525, 1.513); 0.671
	Zanzan	762	108	2.330 (1.476, 3.679); 0.0001
Educational status	No education	7,542	452	1.001 (0.682, 1.471); 0.992
	Primary	2,518	223	1.193 (0.8200, 1.736); 0.356
	Secondary	3,308	282	1.172 (0.828, 1.659); 0.369
	Higher	495	57	1
Religion	Muslim	6,534	389	0.785 (0.621, 0.993); 0.044
	Catholic	2,218	200	0.938 (0.735, 1.197); 0.609
	Evangelical	3,073	269	0.959 (0.764, 1.204); 0.723
	Other	2,038	156	1
Marital status	Never in union	4,211	289	0.909 (0.602, 1.372); 0.649
	Married	4,891	327	0.712 (0.520, 0.975); 0.034
	Living with partner	4,076	330	0.840 (0.616, 1.146); 0.272
	Other	685	68	1
Sex of the household head	Male	10,811	744	1
	Female	3,052	270	1.044 (0.871, 1.250); 0.640
Wealth index	Poorest	2,987	148	1
	Poorer	2,950	192	1.388 (0.931, 2.069); 0.107
	Middle	3,195	244	1.692 (0.932 3.071); 0.083
	Richer	2,527	221	1.865 (0.918, 3.788); 0.084
	Richest	2,204	209	1.986 (0.914, 4.313); 0.083
Frequency of reading newspapers	Not at all	12,569	880	1
	Less than once a week	26	78	1.873 (0.660, 1.154); 0.342
	At least once a week	468	56	1.076 (0.762, 1.518); 0.675
Frequency of listening to the radio	Not at all	9,131	515	1
	Less than once a week	2,686	272	1.388 (1.151, 1.674); 0.001
	At least once a week	2,046	227	1.524 (1.241, 1.871); 0.0001
Frequency of watching television	Not at all	5,189	241	1
	Less than once a week	2,092	208	1.649 (1.156, 2.352); 0.006
	At least once a week	6,582	565	1.313 (0.929, 1.856); 0.122
Frequency of using the internet	Not at all	10,710	646	1
	Less than once a week	366	36	1.347 (0.919, 1.974); 0.127
	At least once a week	1,071	130	1.459 (1.157, 1.841); 0.001
	Almost every day	1,716	202	1.359 (1.081, 1.708); 0.008
History of a terminated pregnancy	No	11,191	744	1
	Yes	2,672	270	1.170 (1.017, 1.376); 0.043
Had health insurance	No	13,119	942	1
	Yes	744	72	0.913 (0.678, 1.229); 0.550
Contraceptive utilization	No method	11,072	713	1
	Modern method	2,380	272	1.213 (1.032, 1.427); 0.019
	other method	411	29	0.841 (0.559, 1.265); 0.407
Being tested for HIV	No	7,979	402	1
	Yes	5,884	612	1.342 (1.149, 1.569); 0.0001
Currently working	No	6,029	406	1
	Yes	7,834	608	0.915 (0.787, 1.064); 0.252
Number of unions	never	4,211	289	1
	Once	8,671	642	1.078 (0.934, 1.245); 0.300
	More than once	981	83	1.232 (0.956, 1.588); 0.106
Community media exposures	No	4,384	191	1
	Yes	9,479	823	0.843 (0.581, 1.224); 0.371
Community wealth quantile	Poor	3,685	202	1
	Middle	4,488	323	0.866 (0.572, 1.311); 0.498
	Rich	5,690	489	0.748 (0.423, 1.320); 0.317

AOR, adjusted odds ratio; CI, confidence interval; others (widowed, divorced, and no longer living together).

## Discussions

The prevalence of STIs among women was 6.82% (95% CI: 6.42%, 7.23%) in the 12 months. This finding was lower than the studies which were conducted in Swaziland (19.4%) (28), eastern India (43.6%) (29), Ethiopia (16.7%) (30), Brazil (20.2%) (31), Uganda (26.0%) (32), and Tanzania (30%) (33). This finding was higher than the studies which were conducted in Hong Kong (2.5%) (34). The variations observed in the study could be attributed to several factors, including the study period, estimation method, sample size, socioeconomic status, and geographic location are the potential causes of these variations. This could be further explained by the fact that the cross-sectional study design may influence the results, as it captures the data at a single point in time and cannot establish causal relationships (35). However, the large sample size utilized in this study strengthens the finding by providing greater statistical power and ensuring a more representative analysis of the population (36). Additionally, participants’ cultural, educational, behavioral, and sociodemographic profiles may influence their health-seeking behaviors regarding STIs (STIs) (37). Furthermore, access to media and health facilities can also play a significant role, as these factors affect individuals’ awareness, knowledge, and ability to seek care (38).

The results of the multivariate analysis showed that among women, STIs were statistically and significantly associated with age group, marital status, religion, media exposure (listening to radio, watching television, and using the internet), history of a terminated pregnancy, contraceptive utilization, and being tested for HIV.

The findings of the study demonstrated that the odds of STIs between the ages of 20 and 34 were more likely compared to women whose ages were between 45 and 49 years old. This finding was concurrent with studies that were in sub-Saharan Africa (39), South Africa (40), Uganda (41), and Bangladesh (42). Young people are at a greater risk of acquiring STIs for several reasons. Young women are often sexually active and may engage in unsafe sexual behaviors, such as having multiple partners, sex without a condom, or sex under the influence of drugs or alcohol, which increases their vulnerability to STIs. Additionally, some young people do not undergo the recommended STI tests, and many are hesitant to discuss their sexual health openly and honestly with a doctor or nurse (43). Furthermore, women at this specific childbearing age (25–34 years) may have a higher coital frequency and have unprotected sex to meet the demands of having children (44, 45).

The odds of STIs among women who were living in Denguele, Montagnes, and Zanzan were more likely relative to women who were living in Abidjan. This might be due to the variations in healthcare facilities, health-seeking behaviors, access to media, socioeconomic and sociocultural factors, knowledge, attitude, and practice toward risky sexual behaviors across the regions, which contribute to the regional differences in STIs in Ivory Coast. This can be more explained by the fact that Abidjan, Ivory Coast's economic capital, has experienced significant urbanization, with its urban population reaching 53.1% in 2023. This urban growth has led to improved infrastructure and services, offering residents better access to healthcare, education, and employment opportunities. In contrast, regions like Denguele, Montagnes, and Zanzan remain less urbanized, facing challenges such as limited healthcare facilities and fewer educational institutions (46–48).

The likelihood of STIs was lower in Muslim women compared to women of other religions. This was supported by the studies that were conducted in Saudi Arabia (49), Since extramarital sex is forbidden in Islam, women believe that religion is protecting them from STIs, which also contributes to the low-risk perception (50–53). Low-risk perceptions about STIs among Muslim women contribute to the low prevalence of STIs due to underreporting, under-detection, and under-documentation of STIs. This could be further explained by Muslim women having poor knowledge regarding STI signs and symptoms, prevention, diagnosis, and treatment, in addition to many misconceptions; negative attitudes toward people infected with HIV/AIDS were common, and attitudes were highly influenced by misconceptions and insufficient knowledge. Women with STIs often face blame and judgment, which discourages them from seeking healthcare due to confidentiality concerns (54).

The odds of STIs among women who were married were less likely relative to women who were others (widowed, divorced, and no longer living together). This finding was in line with studies which were conducted in sub-Saharan Africa (55). This might be because societal norms and expectations related to marriage, which value fidelity and monogamy, are frequently linked to marriage. These social norms have the potential to discourage women from engaging in risky behavior by discouraging extramarital affairs (56). Those social norms, rooted in religious and social beliefs such as the principle of "no sex before marriage" have the potential to discourage women from engaging in risky behaviors like extramarital affairs, thereby contributing to a low incidence of sexually transmitted infections (STIs) among married women (56). Moreover, marriage can foster emotional closeness and fulfillment, which lessens the perception of the need to pursue satisfaction through risky extramarital sex.

The odds of STIs among reproductive-age women who listened to the radio at least once a week; watching television less than once a week; and using the internet almost every day were more likely relative to their counterparts. Were more likely relative to their counterparts. This might be that some media particularly internet-mediated platforms, including social media, video-sharing sites, and online forums, can expose adolescent women to sexually explicit content. This exposure may influence their attitudes, perceptions, and behaviors related to sexual activity, potentially leading to risky sexual behaviors such as early sexual initiation, unprotected sex, and multiple sexual partners (57). Therefore, raising awareness about the proper use of media, particularly internet platforms, could be a key strategy for reducing STIs among women (58–60). In contrast to this finding, several works of literature revealed that media exposure reduces the risks of STIs among women (61–63). This could be because the symptoms of most STIs are subtle and often undetected in women. Therefore, women who had media exposure could get better information and knowledge about STIs, which increases the rate of reporting STIs (64, 65) and early diagnosis of STIs. It offers the best opportunity for effective treatment, preventing complications and reducing the further transmission of STIs (66).

When compared to their counterparts, the likelihood of STIs was higher in women who had a history of a terminated pregnancy. This finding was in line with the studies which were conducted in Ethiopia (67), China (68), Ethiopia (69, 70), and Shandong province of China (71). The scientific explanation for this could be that having a history of abortion may increase women's susceptibility to sexually transmitted infections, especially when performed unsafely by unskilled or traditional practitioners without adherence to aseptic techniques, facilitating STI transmission (26, 72). Furthermore, this might be because the fact that those women who had a history of termination of pregnancy would have better access to reproductive health care services, sexual health services, and a better understanding of the symptoms of STIs, which prevents underreporting and underscreening of STIs and contributes to high detection rates of STIs among women who had a history of a terminated pregnancy.

The odds of STIs among women who use modern contraceptives were higher compared to women who did not use contraceptives. This finding was in line with the studies which were conducted in Bangladesh (42) and India (73). This may be because most contraceptive methods (non-barrier methods) are not highly effective in preventing both pregnancy and STIs. Furthermore, we speculated that many reproductive-age women perceive the risk of pregnancy to be higher than the risk of STIs. As a result, these women often use modern contraceptives, particularly emergency contraceptives, to prevent pregnancy but engage in unprotected or unsafe sexual activities, which contributes to the high incidence of STIs among women. Additionally, the inappropriate use of barrier methods, such as condoms, results in a 21%–40% failure rate in protecting against STIs (74).

STIs among women who were tested for HIV were more likely compared to women who were not tested for HIV. This finding was supported by the studies which were conducted in sub-Saharan Africa (75). Since most STIs are asymptomatic, the reasons for these variations might be that women who were tested for HIV might have more awareness about the symptoms of HIV and other STIs, which results in a high rate of screening and self-reporting of STIs. On the other hand, women who weren’t tested for HIV might not know their status results in low self-reporting of STIs.

## Conclusion

This study found that nearly seven out of a hundred reproductive-age women in Ivory Coast had sexually transmitted infections, influenced by factors such as age group, region, religion, marital status, media exposures, history of a terminated pregnancy, and contraceptive utilization. Therefore, to reduce STIs among reproductive-age women, the governments of Ivory Coast and other concerned stakeholders should give special attention to women whose ages are between 20 and 34 years old, a highly risky region, promoting the media that were broadcasting STI prevention information and creating awareness about STIs for women who had a history of a terminated pregnancy and who used modern contraceptive methods.

### Strengths and limitations of this study

This study has several strengths, including the standardized Demographic and Health Survey (DHS) design and a large, representative sample which enhances the generalizability of the findings across the diverse populations. The use of nationally representative data improves the reliability of estimates and allows for a comprehensive understanding of factors associated with STIs among reproductive-age women in Ivory Coast.

However, certain limitations should be considered. Recall bias may be present in retrospective data, as participants may not accurately remember past events, making it difficult to ensure data accuracy. Additionally, the cross-sectional study design limits the ability to establish temporal or causal relationships between predictor variables and STIs among women, restricting the ability to determine whether specific predictors directly contribute to STI acquisition. Furthermore, reliance on self-reported STIs without laboratory confirmation may introduce reporting bias, as some participants may underreport or overreport their condition due to stigma, lack of awareness, or misunderstanding of symptoms. Despite these limitations, the study provides valuable insights into the factors associated with STIs among reproductive-age women and the need for targeted public health interventions to reduce the burden of STIs in this vulnerable population.

## Data Availability

Publicly available datasets were analyzed in this study. This data can be found here: https://dhsprogram.com/data/dataset_admin/index.cfm.
